# Interesting Case of Skull Injury

**Published:** 1884-08-20

**Authors:** Wm. Perrin Nicolson

**Affiliations:** Professor of Anatomy in Southern Medical College, Atlanta, Ga., and Surgeon to Ivy Street Hospital


### Southern Medical Record:

EDITORS:
THOMAS S. POWELL, M.D.     R. C. WORD, M.D.

*R. C. WORD, M.D., Managing Editor.*

☞All Communications and Letters on Business connected with the RECORD must be addressed to the Managing Editor.

VOL. XIV.   ATLANTA, GA., AUGUST 20, 1884.   No. 8

### ORIGINAL AND SELECTED ARTICLES.

### INTERESTING CASE OF SKULL INJURY.

BY WM. PERRIN NICOLSON, M.D.,
Professor of Anatomy in Southern Medical College, Atlanta, Ga., and
Surgeon to Ivy Street Hospital.

Robert Turner, colored, aged about fifty-five, was admitted to Ivy Street Hospital May 20th, 1884, with the following history: Two days before he had been struck upon the head with a rock thrown by a negro boy, causing him to fall immediately in an unconscious condition. He was placed in a conveyance and brought to the police station, where he lay during the day following, which was Sunday. He was seen during that day by Dr. Kerstan, ward physician, and on Monday was admitted to the Hospital. When called to see him I found him lying unconscious, with stertorous breathing. There was hemiplegia of the right side, partial in the upper, and complete in the lower extremity. The pupils were about normal in dimension, though not reacting to light. The pulse was very full and slow. Having been informed that the brain injury was caused by a blow, I made an examination of the head, which was rendered easy by the patient being very bald. Only after the location was pointed out to me precisely did I detect over the left parietal bone, just behind the coronal suture, a mere abrasion of the skin, not sufficient to bring blood. By pressing over this point a sharp depression was found, seemingly caused by some sharp instrument. There was no decided contusion of the scalp. The only voluntary motion of the patient was carrying the left hand continually to this spot.

The question to be decided was the indication for or against trephining, and it was thought best to await developments. The patient was given a full dose of calomel; cold was applied to the head; and a mixture containing bromides with ergot given every two hours. The next day, as his condition was somewhat better, it was decided not to entertain the idea of trephining unless there was a serious change for the worse. He continued in about the same condition that day, when, during the night, he suddenly commenced to sink and died in a few hours.

As there was some delay in the coroner's inquest, and no examination was made beyond cutting down upon the fracture, the post mortem examination was not made for more than forty-eight hours. At this time there was marked progress of decomposition, and, upon opening the wound, gas was noticed escaping from the cranial cavity. The scalp was then dissected away, showing contusion, and effusion of some blood about the site of the injury. The skull was found depressed over an oval space about one by two inches, with a sharp depression in the centre. Removing the skull, a scale of the inner table was left adherent to the dura mater. There were no spiculæ about the fracture. A small clot of blood was found between the separated inner table and the dura mater. Opening the dura mater, the right hemisphere was found deeply congested over its whole surface, and a considerable amount of blood was effused over the arachnoid membrane. Still not finding enough blood to account for the great compression, I incised the anterior lobe of the brain, when a clot almost as large as a small hen's egg was found, surrounded by softened blood and brain tissue.

Thus, it was decidedly proven that trephining could have resulted in no possible good, and would only have assisted in bringing discredit upon the operation. That so extensive injury of the brain structures could have resulted from a blow which produced so little external impression is somewhat remarkable, and the case goes to show conclusively that trephining is not to be unadvisedly undertaken, no matter how favorable the apparent indications may be. It also shows that in such instances little idea can be formed of the actual extent of injury within the cranial cavity.

